# Assessment of psychopathological symptoms in patients with primary hypothyroidism

**DOI:** 10.1192/j.eurpsy.2022.615

**Published:** 2022-09-01

**Authors:** O. Pityk, N. Karbovskyi

**Affiliations:** Ivano-Frankivsk National Medical University, Psychiatry, Narcology And Medical Psychology, Ivano-Frankivsk, Ukraine

**Keywords:** Hypothyroidism, nonpsychotic mental disorders, psychopathological symptoms

## Abstract

**Introduction:**

Thyroid dysfunction such as hypothyroidism, is connected with numerous neurological and psychiatric disorders. However, the importance of assessing the interaction between brain, psyche and thyroid in clinical practice is often underestimated, and this has a direct impact on the planning of therapeutic interventions and treatment efficacy in patients with primary hypothyroidism.

**Objectives:**

We examined 132 patients with primary hypothyroidism.

**Methods:**

Assessment of the presence and severity of psychopathology was performed using the technique SCL-90-R (questionnaire severity of psychopathology).

**Results:**

The results showed the highest scores on the scales of somatization (3,75 ± 0,12), depression (3,64 ± 0,13), interpersonal anxiety (3,45 ± 0,19), phobias (3.25 ± 0,31). High rates of somatization scale showing a violation of bodily dysfunction of various body systems-cardiovascular, gastrointestinal, respiratory and headache, muscular discomfort and other unpleasant sensations in different parts of the body and manifest themselves in a complaint of patients. Scale depression revealed the presence of dysphoria, anhedonia, low affect, loss of vitality and interest in life. Relatively high on a scale of phobias indicate the presence in these patients persistent fear responses to certain situations and objects that are irrational and inadequate and lead to avoiding behavior. General index of severity of symptoms (GSI) and the index of an existing symptomatic distress (PSDI) were significantly higher in the following patients than in hypothyroid patients without mental disorders.

**Conclusions:**

Thus, these results should be taken into account when determining treatment strategy both in psychopharmacotherapy and different methods of psychological correction.

**Disclosure:**

No significant relationships.

